# Microvascular Dysfunction in Hypertrophic Cardiomyopathy

**DOI:** 10.3390/jcm11216560

**Published:** 2022-11-04

**Authors:** Francesco Pelliccia, Franco Cecchi, Iacopo Olivotto, Paolo G. Camici

**Affiliations:** 1 Department of Cardiovascular Sciences, Sapienza University, 00166 Rome, Italy; 2 IRCCS Istituto Auxologico Italiano, Department of Cardiovascular, Neural and Metabolic Sciences, San Luca Hospital, 20100 Milan, Italy; 3 Department of Experimental and Clinical Medicine, University of Florence, Meyer Children Hospital and Careggi University Hospital, 50123 Florence, Italy; 4 San Raffaele Hospital, Vita-Salute University, 20121 Milan, Italy

**Keywords:** hypertrophic cardiomyopathy, microcirculation, myocardial ischemia

## Abstract

Myocardial ischemia is an established pathophysiological feature of hypertrophic cardiomyopathy (HCM) that impacts various clinical features, including heart failure (HF) and sudden cardiac death (SCD). The major determinant of myocardial ischemia in HCM is coronary microvascular dysfunction (CMD) in the absence of epicardial coronary artery abnormalities. Despite the impossibility to directly visualize microcirculation in vivo, a multimodality approach can allow a detailed assessment of microvascular dysfunction and ischemia. Accordingly, the non-invasive assessment of CMD using transthoracic Doppler echocardiography, positron emission tomography, and cardiac magnetic resonance should now be considered mandatory in any HCM patient. Noteworthy, a complete diagnostic work-up for myocardial ischemia plays a major role in the approach of the patients with HCM and their risk stratification. Chronic and recurrent episodes of ischemia can contribute to fibrosis, culminating in LV remodeling and HF. Ischemia can potentially constitute an arrhythmic substrate and might prove to have an added value in risk stratification for SCD. Accordingly, strategies for the early diagnosis of CMD should now be considered an important challenge for the scientific community.

## 1. Introduction

Myocardial ischemia is an established pathophysiological feature of hypertrophic cardiomyopathy (HCM) that impacts various clinical features, including heart failure (HF) and sudden cardiac death (SCD). Since the original demonstration in the 1980s that perfusion defects can be found in over half of patients [1,2], myocardial ischemia has been recognized as a common complication of the disease due to the multiple potential factors that may contribute to the inadequate oxygen supply and the higher susceptibility for myocardial hypoxia. First, the cardiomyopathic heart is characterized per se by an increased oxygen demand due to the larger myocardial mass and increased filling pressures [3,4,5]. Additionally, regional blood flow may be reduced by concomitant epicardial coronary artery disease (CAD), which can be detected in around 10% of patients and is a marker of poor prognosis in HCM [6]. Furthermore, there is growing evidence that anomalous coronary anatomy and myocardial bridges are common in HCM and may contribute to ischemia by prolonged coronary compression and reduced blood flow in the early diastolic phase [7]. The major pathophysiologic determinant of myocardial ischemia in HCM, however, is coronary microvascular dysfunction (CMD) in the absence of epicardial coronary artery abnormalities [8,9,10].

This review aims to outline the contemporary diagnostic work-up for myocardial ischemia in patients with HCM, followed by exploring the main clinical implications of CMD as a potential pathophysiologic determinant of heart failure (HF) or a predictor of increased risk of arrhythmic complications and sudden cardiac death (SCD).

## 2. Coronary Microvascular Dysfunction in HCM

**Figure 1:**
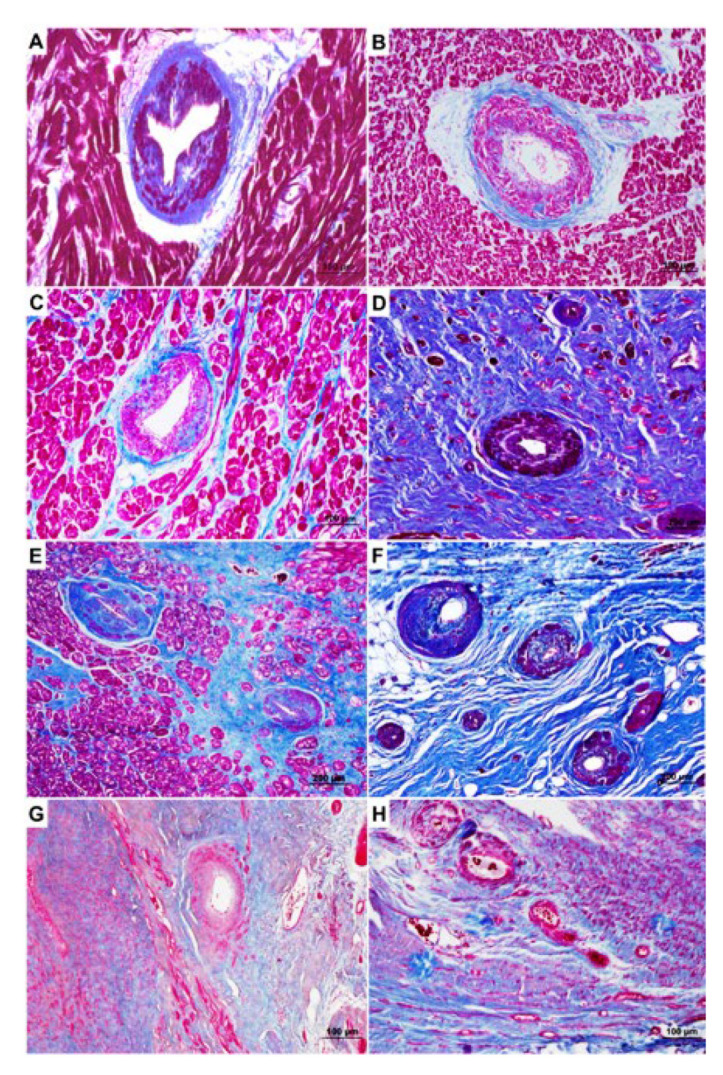
Pathologic appearance of small vessel disease (SVD) in HCM. (**A**,**B**) HCM patient with SVD in the absence of replacement-type fibrosis. (**C**,**D**) SCD-HCM patient with SVD in areas without (**C**) and with (**D**) fibrosis: note the higher score in the area with replacement-type fibrosis. (**E**,**F**) HCM patient with SVD in areas without (**E**) and with (**F**) fibrosis. (**G**,**H**) Ischemic heart disease patient showing large areas of replacement-type fibrosis with SVD, with a lower score as compared to HCM cases. (**A**–**H**) Heidenhain trichrome. The figure is from De Gaspari M et al., with permission [11].

CMD in HCM has a multifactorial origin, including either ultrastructural abnormalities and hemodynamic factors. The pathologic bases are the anatomic changes of the small intramural coronary arteries, including medial hypertrophy, intimal hyperplasia, and decreased luminal size. The underlying pathological changes of CMD involve medial hypertrophy, intimal hyperplasia, and decreased luminal size of the small intramural coronary arteries (Figure 1) [11]. The hemodynamic factors include some of the functional characteristics of the disease, i.e., extravascular compression due to ventricular hypertrophy, diastolic dysfunction, and LV outflow tract obstruction [10]. Of note, CMD causes a reduction in coronary vasodilator reserve that is not confined to the hypertrophied regions of LV myocardium but is extended to the entire ventricle, pointing to a primary involvement. Interestingly, impaired myocardial oxygenation has been detected in carriers of HCM mutations prior to development of LV hypertrophy, suggesting that the microcirculation may be affected early in the disease process [12,13,14]. Under normal circumstances, the small coronary arterioles <450 μm in diameter are the principal determinants of coronary vascular resistance [15]. Half of the total coronary vascular resistance is located in pre-arterioles >100 μm, which are innervated by the autonomic nervous system [16]. Nearly all of the remaining vascular resistance lies in vessels <100 μm in diameter, which are also those responsible for the auto-regulation of blood flow [16]. In addition to intravascular resistance, myocardial perfusion is also influenced by extravascular forces, particularly due to the intra-myocardial pressure which is generated throughout the systolic phase [15]. The intra-myocardial pressure is maximal during systole and in the sub-endocardial layers where it exceeds the aortic pressure [15]. Although direct visualization of the coronary microcirculation has been achieved in experimental models, the study of the human coronary microcirculation is indirect and relies on the assessment of parameters which reflect its functional status, such as measurement of myocardial blood flow (MBF) and coronary flow reserve (CFR). Unfortunately, these parameters are not routinely assessed in patients with HCM in clinical practice. As a consequence, no data on the prevalence of CMD in ‘real world’ HCM patients are available at present.

## 3. Diagnosis of Coronary Microvascular Dysfunction in HCM

Current evidence indicates that investigation of myocardial ischemia should become a crucial part of the diagnostic work-up of all patients with HCM regardless of clinical history and presentation. Typical and atypical chest pain is a frequent symptomatic feature in HCM, but myocardial ischemia can also occur silently. A study performed in asymptomatic patients with HCM demonstrated reversible perfusion defects, indicative of ischemia in 50% of cases [17]. Although signs of inducible ischemia may be detected by electrocardiography, echocardiography, or myocardial scintigraphy, an accurate quantitative assessment of myocardial ischemia due to CMD is not easily feasible in clinical practice.

Doppler echocardiography has a pivotal role in the diagnostic work-up of patients with HCM as the technique is virtually available worldwide. It is a noninvasive method that allows the study of hypertrophy distribution and quantification of the wall thickness, LV outflow tract obstruction, and diastolic function [18]. The ability of rest or stress echocardiography to identify epicardial CAD is limited, as exercise induced wall motion abnormalities in HCM have a complex and multifactorial pathophysiology and therefore are not good predictors of epicardial CAD [19] With adenosine, however, CFR can be assessed either in the left anterior descending artery or in the posterior descending artery [20]. Using Doppler echocardiography for the evaluation of microcirculation, a greater diastolic flow velocity and lower CFR has been observed in HCM patients compared to normal subjects [21]. Doppler echocardiography is operator dependent, suffers from significant intra-observer and inter-observer variability, and may not provide reliable results in case of obesity and lung disease.

Myocardial perfusion imaging with single-photon-emission computed tomography (SPECT) can define the presence and severity of ischemia. Historically, the concept of myocardial ischemia in HCM was originally introduced using thallium-2011 in pivotal studies that showed its relation to adverse events [22,23]. At present, SPECT has a limited diagnostic value since quantification of flow cannot be obtained.

**Figure 2:**
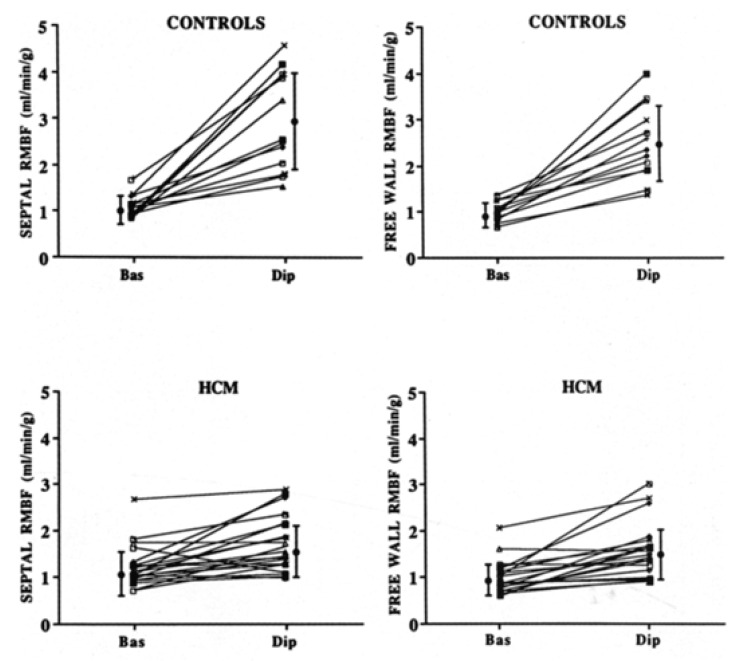
Both microvascular ischemia and replacement fibrosis can be evaluated by CMR, the former by first-pass perfusion and the latter by late gadolinium enhancement (LGE). Importantly, there is a correlation between LGE and the grade of hyperemic MBF assessed by PET, thus suggesting that LGE corresponds—at least in part—to replacement fibrosis secondary to microvascular dysfunction and myocardial ischemia. The figure is from Camici PG et al., with permission [25].

Positron emission tomography (PET) allows us to measure MBF quantitatively, both at rest and after vasodilation, and, consequently, to calculate the CFR. Camici et al., first described an inadequate increase in MBF following the intravenous administration of dipyridamole in the majority of HCM patients studied with PET (Figure 2) [8]. Of note, microvascular dysfunction seems to be more pronounced in hypertrophied segments and in the subendocardial layers, particularly in patients with LV systolic dysfunction [24]. However, it may also be found in non-hypertrophied segments, suggesting a diffuse impairment in coronary microvascular function [25]. Interestingly, when assessed by PET, the difference in MBF between endocardium and epicardium after dipyridamole administration was reversed after treatment with verapamil in one study [26].

Myocardial ischemia due to CMD can be detected by cardiac magnetic resonance (CMR) as well. CMR has emerged as an ideal complementary technique to echocardiography since it provides a detailed characterization of HCM morphology, can detect the presence of fibrosis, and allows assessment of functional features, particularly abnormalities of papillary muscles and mitral apparatus [27,28]. Both microvascular ischemia and replacement fibrosis can be evaluated by CMR, the former by first-pass perfusion and the latter by late gadolinium enhancement (LGE). Importantly, there is a correlation between LGE and the grade of hyperemic MBF assessed by PET, thus suggesting that LGE corresponds—at least in part—to replacement fibrosis secondary to microvascular dysfunction and myocardial ischemia [29].

Coronary circulation in HCM can be demonstrated invasively by means of Doppler flow velocity guidewires and, more recently, by combined pressure and temperature guidewires that allow simultaneous measurement of coronary pressure and flow, thus potentially facilitating the choice of optimal medical therapy similarly to what occurs in other subsets of patients [30]. Overall, MBF studies have shown a blunted CFR coupled with lower coronary resistance compared with controls, thus indicating that reduction in CFR in HCM is secondary to near-maximal coronary vasodilation due to the higher demand imposed by the increased LV mass [31].

## 4. Clinical Implications of CMD in HCM

The identification of patients at risk for sudden cardiac death (SCD) or progression to heart failure (HF) constitutes one of the main clinical concerns in HCM [32,33,34,35,36]. The role of myocardial ischemia as a predictive factor should be always taken into consideration in HCM [32] as it may be associated with important complications that impact clinical outcome [31]. Similar to what occurs in patients with CAD, there is now agreement that chronic or recurrent ischemic injury might promote deposition of collagen leading to replacement fibrosis, a pathologic finding distinct from the progressive reactive interstitial fibrosis which is typically found in HCM [37]. Indeed, CMD may lead over time to recurrent ischemia and myocyte death, thus acting as a localizer of replacement fibrosis. The clinical consequences are either adverse LV remodeling and HF or arrhythmias and SCD.

### 4.1. CMD and Risk of Heart Failure

Multiple prospective investigations have clearly demonstrated that patients with HCM are prone to developing heart failure (HF) [38,39,40]. Most importantly, those who experience HF have a high risk of death from both progressive pump failure and SCD and have an annual mortality ten-fold higher than the general HCM population [41,42,43].

**Figure 3:**
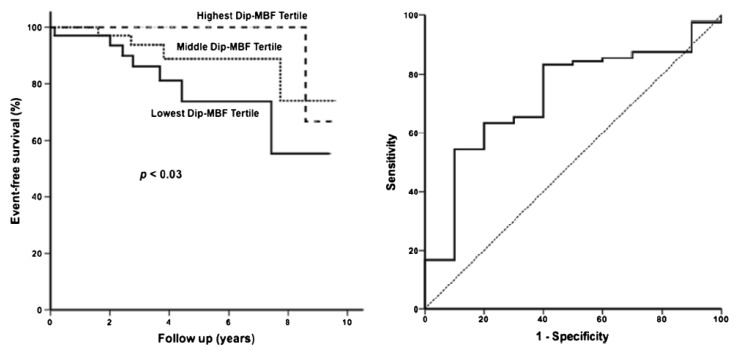
Myocardial blood flow (MBF) values after dipyridamole infusion and long-term prognosis. Survival analysis showed a significant increase in risk associated with lower Dip-MBF tertiles (right panel). ROC curve analysis identified a Dip-MBF < 1.35 mL/min/g as the best threshold for prediction of and unfavorable outcomes (left panel). The figure is from Castagnoli H et al., with permission [45].

The prognostic role of myocardial ischemia has clearly been demonstrated in a study by Cecchi et al., who showed that the degree of CMD is a strong, independent predictor of clinical deterioration and death [44]. An age-adjusted multivariate analysis demonstrated that a low dipyridamole MBF was the most powerful independent predictor of outcome, with a 9.6 times increased risk of cardiovascular mortality for patients in the lowest tertile (i.e., with a dipyridamole flow ≤1.1 mL/g/min). Specifically, all the four heart-failure-related deaths and three of five SCDs occurred among the 18 patients in the lowest tertile of dipyridamole flow [44]. It is noteworthy that at the time of the PET scan, none of the patients had severe symptoms, and only a few would have been considered at high risk on the basis of the established indicators of outcome. Nevertheless, substantial microvascular dysfunction could already be demonstrated, several years before their clinical progression, in most of those patients who subsequently deteriorated or died [44]. The value of maximal MBF value was recently confirmed in a recent study on a larger HCM population, with a slight increase level to 1.35 mL/min/g (Figure 3) [45]. These results have intriguing implications in that PET evaluation of MBF may significantly improve risk stratification and allow the implementation of preventive measures in clinically stable patients with HCM [46]. Indeed, a severe CMD—as indicated by a MBF of 1.1 mL/min/g or lower after dipyridamole infusion—can be considered a strong predictor of unfavorable outcome and cardiovascular mortality [46]. Of note, these findings expand upon previous observations that the detection of microvascular abnormalities in HCM allows us to discriminate between the disease and athletes with LV hypertrophy [47].

### 4.2. CMD and Risk of Sudden Cardiac Death

Myocardial ischemia is now thought to be responsible for some of the most worrisome complications of HCM, which are malignant arrhythmias and SCD [48,49]. Autopsy findings from individuals with HCM-related SCD found a high prevalence of histological changes consistent with acute or subacute myocardial ischemia, possibly forming the substrate for arrhythmogenesis [50]. Pivotal studies with SPECT suggested an association between myocardial ischemia, syncope, and SCD [22]. Flow heterogeneity using positron emission tomography (PET) has been associated with ventricular arrhythmias [51], suggesting a potential role for PET in SCD risk assessment. More recently, CMR has demonstrated reversible and fixed uptake defects without obstructive CAD on invasive coronary angiography, suggesting ischemia and scar, respectively [52,53].

Despite the long-standing evidence that myocardial ischemia can play a pathogenetic role in SCD, the 2014 European guidelines failed to include assessment of myocardial ischemia in the risk score for prediction of SCD. The score includes only some factors already considered in previous documents (maximum LV wall thickness, family history, and syncope) with the addition of three continuous variables, i.e., age, LV outflow tract gradient, and left atrial diameter [54]. It is tempting to speculate that the limitations of this score might have played a role in frequency of SCD in later years. Although the incidence of SCD is now lower than previously reported in the original series of the 1980s and 1990s, most SCDs occur in patients who have not been deemed to be at high risk and have not been treated with a defibrillator [55].

The highly anticipated American Heart Association/American College of Cardiology (AHA/ACC) HCM guidelines have been recently released [56]. The update lists 133 recommendations for HCM care in several categories, including role of high-volume HCM centers, diagnosis, initial evaluation and follow-up, risk assessment and prevention of SCD, and lifestyle considerations. As regards risk factors for SCD, the 2020 American document highlights the fact that retrospective observational studies over the past decade have identified a number of noninvasive clinical risk markers associated with increased risk for SCD in HCM. In association with clinical judgment and shared decision-making, patients with HCM are considered potential candidates for primary prevention indication to implantable cardioverter defibrillators if they have ≥1 of the traditional major risk markers, i.e., family history of SCD, massive LV hypertrophy, and unexplained syncope, or one novel marker, such as apical aneurysm, LGE on CMR, and impaired LV systolic function (ejection fraction<50%). This choice is based upon the evidence that large areas of myocardial fibrosis (>15% of LV mass)—which now can be easily detected by LGE on CMR—are associated with an increase in arrhythmic events resulting from a reentry circuit mechanism [57].

LV apical aneurysm represents a further important example of the pro-arrhythmic effects that myocardial ischemia unrelated to CAD might have in HCM. It is now said that apical aneurysms showing an obstructive pattern are areas of myocardial scarring caused by continuous exposition of the apical myocardium to high LV wall stress and systolic pressures, which in turn lead to pressure overload, greater oxygen demand, abnormal coronary perfusion, and myocardial ischemia [57]. Apical aneurysms constitute a potential site of anatomic reentry for sustained monomorphic ventricular tachycardia at the junction between the scarred aneurysm rim and adjacent viable myocardium mechanism [58]. Interestingly, Maron et al., incorporated LGE and apical aneurysm in a risk factor prediction algorithm and were able to identify almost 95% of HCM patients at risk of SCD [59].

In summary, the current consensus is that ventricular arrhythmogenesis and the risk of SCD in HCM clearly relates to the combination of abnormal cellular substrate, ventricular anatomy, dynamic changes in hemodynamics, rhythm disturbances, and myocardial ischemia [32,60].

## 5. Treatment of CMD

Although myocardial ischemia has a well-recognized pathogenetic role in HCM, the best pharmacologic treatment of CMD has yet to be identified. From a theoretical point of view, however, pharmacologic agents that have shown to exert favorable effects on CMD in patients with CAD have the potential to be effective in primary cardiomyopathies as well.

Angiotensin-converting enzyme (ACE) inhibitors have been proposed for the treatment of CMD in patients with non-obstructive CAD, based on pleiotropic actions on the vascular wall (i.e., reverse remodeling of intramural coronary arterioles) and consequent improvement in CFR [61]. However, it is important to underline the fact that ACE inhibitors should be used with caution in HCM as they may be harmful in the subset of patients with LV outflow tract obstruction due to their vasodilating effects.

Calcium channel blockers in general, and dihydropyridines in particular, are potentially useful to treat CMD due to their vascular relaxing effect and dilator effect on coronary resistant vessels [62]. Nevertheless, although verapamil is often used in patients with HCM, no previous investigation has demonstrated any clinical effect on microcirculation.

Late sodium current inhibitor ranolazine has been reported as an effective drug to control angina symptoms in several cardiac conditions, including HCM patients [33,63]. However, the RESTYLE-HCM randomized, placebo-controlled trial failed to show any significant improvement with ranolazine on functional capacity, symptomatic status, and diastolic function, with only a decrease in the number of ventricular arrhythmias being associated with ranolazine [64].

At variance with pharmacologic agents that seem to be unable to improve myocardial perfusion in HCM [65,66,67,68], mechanical reduction of LV outflow tract gradient by alcohol septal ablation or surgical myectomy seem to improve CFR and septal endocardial-to-epicardial MBF [69,70]. The evidence that only invasive strategies aimed at reducing or abolishing LV obstruction have been shown to affect CMD in patients with HCM suggests that further investigations on pharmacologic options are needed. As a matter of fact, these patients suffer from ischemia-related symptoms and might experience major adverse cardiac events. Thus, large outcome trials testing the efficacy of currently available traditional anti-atherothrombotic and anti-ischemic therapy, as well as novel therapies in this population, are warranted.

## 6. Conclusions

**Figure 4:**
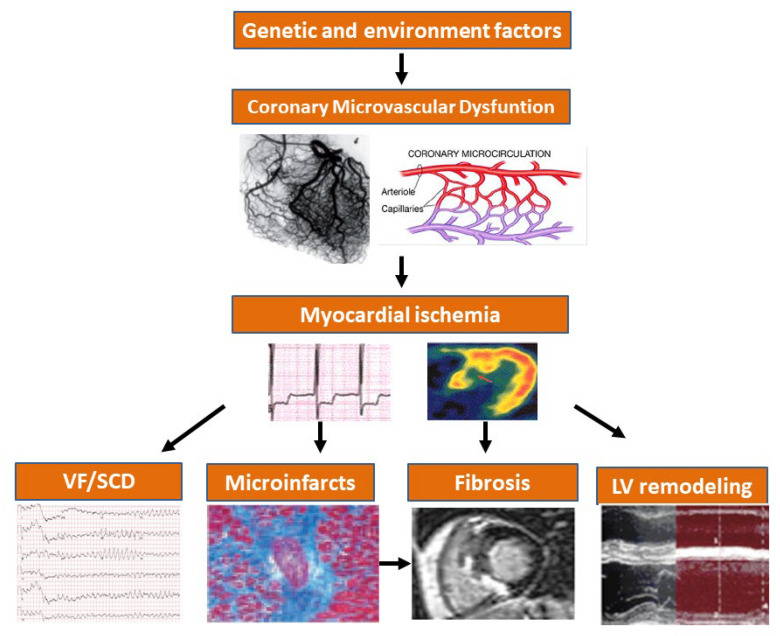
Proposed cascade of events in hypertrophic cardiomyopathy. Genetic and environmental factors cause abnormal coronary arteriolar remodeling and microvascular dysfunction leading to myocardial ischemia and fibrosis. The clinical consequences are either adverse left ventricular remodeling and heart failure or arrhythmias and sudden cardiac death.

Myocardial ischemia secondary to CMD is a major pathophysiological feature of HCM, that might impact various pathological and clinical features encompassing tissue abnormalities and arrhythmic events (Figure 4). A comprehensive diagnostic work-up plays a major role in the approach of the patients with HCM and their risk stratification [60] as the non-invasive assessment of CFR using transthoracic Doppler echocardiography, PET, and CMR is now mandatory in any HCM patient. Guidelines on HCM have been revised and updated in light of most recent evidence but have not clearly acknowledged the emerging role of well-recognized predictive factors such as myocardial ischemia [7,8]. While unraveling the prognostic role of genetics, clinical and preclinical features potentially associated with a higher risk of HF and SCD such as CMD now have the potential for improving risk stratification in patients with HCM.

## Data Availability

Article did not report any data.
